# All in the Family: Multi‐generational support for families affected by rare dementia

**DOI:** 10.1002/alz70858_104605

**Published:** 2025-12-26

**Authors:** Mary Pat Sullivan, Veronika Williams, Jeffery Thornborrow

**Affiliations:** ^1^ Nipissing University, North Bay, ON, Canada

## Abstract

**Background:**

Much of our knowledge on the psychosocial impact and care needs of people living with dementia and interventions to improve well‐being normally focus on a principal caregiver – namely a spouse or adult child. Gaps in the literature include understandings of the impact on younger children or grandchildren, siblings, parents and parents‐in‐law or close friends. Knowledge on how to support the entire family system is also scarce yet highly relevant in the instance of an atypical or rare dementia diagnosis which often affects people at a young age. The aims of this proof‐of‐concept study were to: (a) develop understandings of the impact of rare dementia on the multiple generations and relationships within a family system; (b) what characterizes a model of multi‐ or intergenerational support; and (c) pilot a novel data collection method with youth and young adults.

**Method:**

Rare Dementia Support Canada members to were invited to participate. Our sample included spouses and parents of people living with young onset dementia (*N* = 9) who participated in an in‐depth interview to explore the emotional, practical and informational needs of children and parents. We also engaged youth aged 12‐17 (*N* = 3), young adults aged 18‐24 (*N* = 5) who responded to questions about daily life with a parent with young onset dementia via Instagram's direct messaging using text, video or audio. These participants were also involved in a virtual group interview to learn about their experiences using Instagram. All data was analyzed using reflexive thematic analysis.

**Result:**

Our results point to the absence of support for the entire family system or those tailored to individual members of the family such as children, siblings, parents, or close friends. From the perspective of children and parent participants, stories were balanced among those of grief, loss, interruptions in developmental transitions and those of connection, memory making and normalization. For parents there was a significant desire to learn how to help. Our use of Instagram provided varied data quality though was viewed as a convenient and accessible way to participate in research.

**Conclusion:**

Using participatory approaches, this study is currently informing the development of a larger investigation into family‐centred rare dementia support.

